# Misdiagnosis of Susac syndrome as demyelinating disease and primary angiitis of the central nervous system: A case report

**DOI:** 10.3389/fneur.2022.1055038

**Published:** 2022-12-08

**Authors:** Gang Wu, Wei Jiang, Zunbo Li, Dehui Huang, Lei Wu

**Affiliations:** ^1^Department of Neurology, The First Medical Centre of Chinese PLA General Hospital, Beijing, China; ^2^Department of Neurology, No 984 Hospital of PLA, Beijing, China; ^3^Netherlands Institute for Neuroscience, Royal Netherlands Academy of Arts and Sciences, Amsterdam, Netherlands; ^4^Department of Neurology, Xi'an Gao Xin Hospital, Xi'an, China

**Keywords:** Susac syndrome, demyelinating, primary angiitis of the central nervous system, acute disseminated encephalomyelitis, encephalopathy

## Abstract

Susac syndrome (SuS) is a rare neuroinflammatory disease that manifests with a triad of hearing loss, branch retinal artery occlusions, and encephalopathy. Patients with SuS are frequently misdiagnosed because the clinical trial is incompletely present at disease onset. In this report, we present a case of a 29-year-old man manifesting sleepiness, epilepsy, urinary dysfunction, and hemiparesis at the initial stage. Magnetic resonance imaging (MRI) revealed multiple abnormal signals located in the lateral paraventricular, corpus callosal, and pons. In addition, the patient had sustained elevation of CSF pressure and protein. ADEM was considered according to the clinical and radiographic findings. However, symptoms were not significantly improved after methylprednisolone therapy. He showed a vision decline in the third month after the disease onset. It was considered from intracranial hypertension or optic neuritis, and therefore retinal arteriolar impairment was ignored. As the disease progresses, cognitive decline was presented. Brain MRI exhibits multiple significant hyperintensities on the DWI sequence with speck-like gadolinium enhancement. Thus, PACNS was diagnosed. The SuS was not made until the presence of hearing decline in the 4 months after the disease onset. The case will be helpful for clinicians to better recognize the atypical initial manifestation of SuS.

## Introduction

Susac syndrome (SuS) is a rare immune-mediated disease that predominantly involves the small arteries of the brain, retina, and inner ear, leading to endothelial cell damage and consequent vascular occlusion. It is characterized by acute encephalopathy, visual impairment, and sensorineural deafness. The disease mainly affects young women (male-to-female ratio of 1:3.2) (1). The rates of misdiagnosis are high, especially when rare clinical manifestations, including epilepsy (4%), urinary dysfunction (9%), and hemiparesis (20%), occur (2). In this report, we present a case of a man with SuS showing these rare symptoms at the early stage of the disease, which was initially thought to be acute disseminated encephalomyelitis (ADEM) and subsequently considered to be primary angiitis of the central nervous system (PACNS).

## Case description

**Figure 1:**
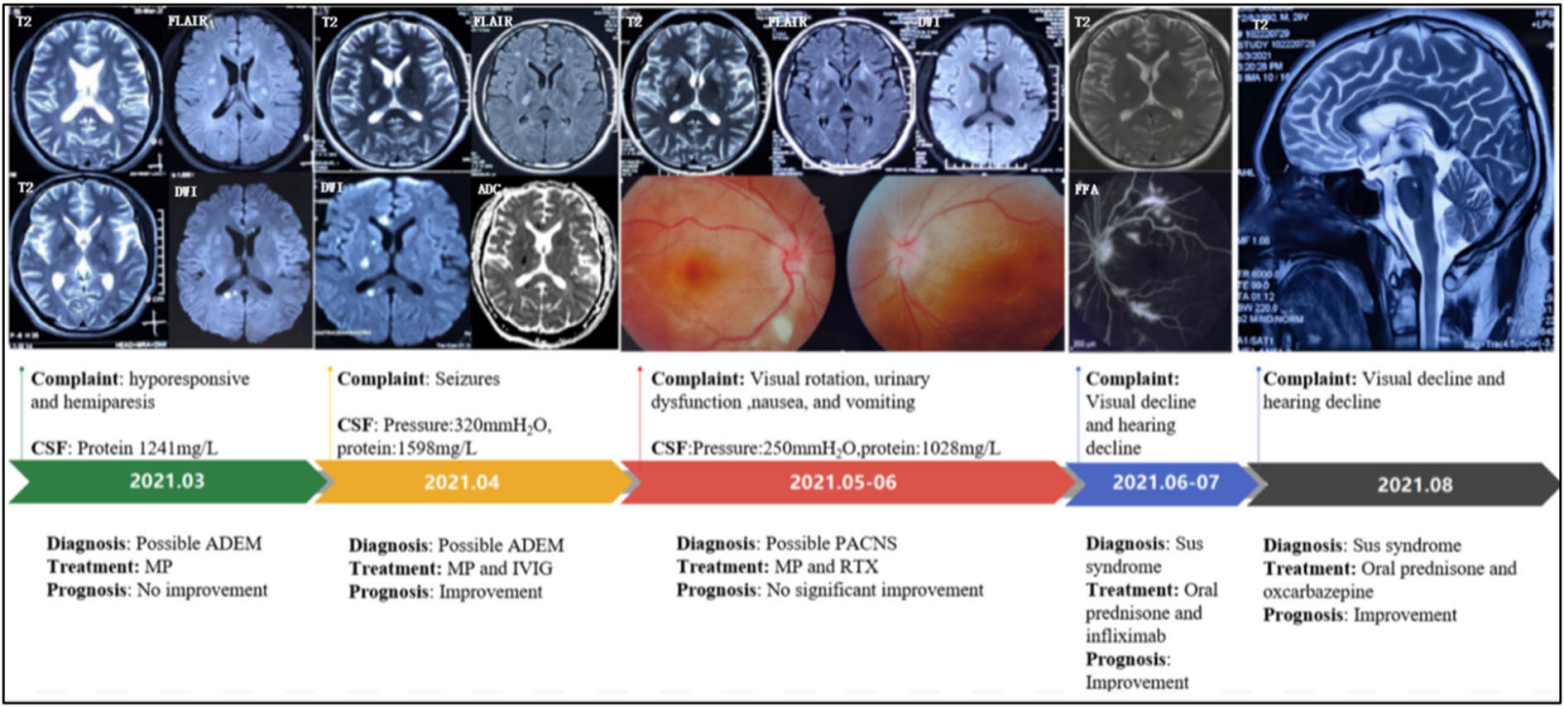
Disease progression map. CSF, cerebrospinal fluid; ADEM, acute disseminated encephalomyelitis; MP, methylprednisolone; IVIG, intravenous immunoglobulin; PACNS, primary angiitis of the central nervous system; RTX, rituximab; FFA, Fundus fluorescein angiography.

A 29-year-old man visited the local hospital because of 3 months of sleepiness, hyporesponsive, dizziness, and left hemiparesis. Physical examination showed that the muscle strengths of the left and right limbs were 4/5 and 5/5, respectively. Brain magnetic resonance imaging (MRI) showed hyperintensities on T2-weighted imaging and sagittal fluid-attenuated inversion recovery imaging (subcortical, periventricular, corpus callosum, and brainstem), with a quasi-circular region of restricted diffusion (Figure 1). Cerebrospinal fluid (CSF) test results revealed 7 × 10^6^/L white-cell count and 4.1 mmol/L glucose, 119.2 mmol/L chloride, and 1,241 mg/L protein levels. No antibodies against AQP4, MOG, GFAP, oligoclonal bands (OBs), autoimmune encephalitis-associated proteins, and paraneoplastic antibodies (anti-Hu, anti-Yo, anti-Ma2, anti-amphiphysin, anti-NMDAR, anti-VGKC, and anti-GABABR) were detected in the serum and CSF. Methylprednisolone was administered after diagnosing the case as possible acute disseminated encephalomyelitis (ADEM). However, the patient subsequently developed slurred speech and had simple-partial seizures (three times daily). Electroencephalogram exhibited median-high–amplitude (theta-delta) wave in the bilateral cerebral hemisphere. A re-examination using MRI revealed enlarged foci without gadolinium-enhanced lesions. CSF test results showed elevated initial pressure (320 mm H_2_O) and protein level (1,598.9 mg/L). Moreover, the results of cell counts (0 × 10^6^/L), smear analysis, and metagenomic next-generation sequencing revealed no abnormalities. After 5 days of immunoglobulin and methylprednisolone treatment, the symptoms were relieved. Brain MRI showed lesion shrinkage. The patient was prescribed low-dose oral prednisone and antiepileptic drugs post-discharge.

**Figure 2:**
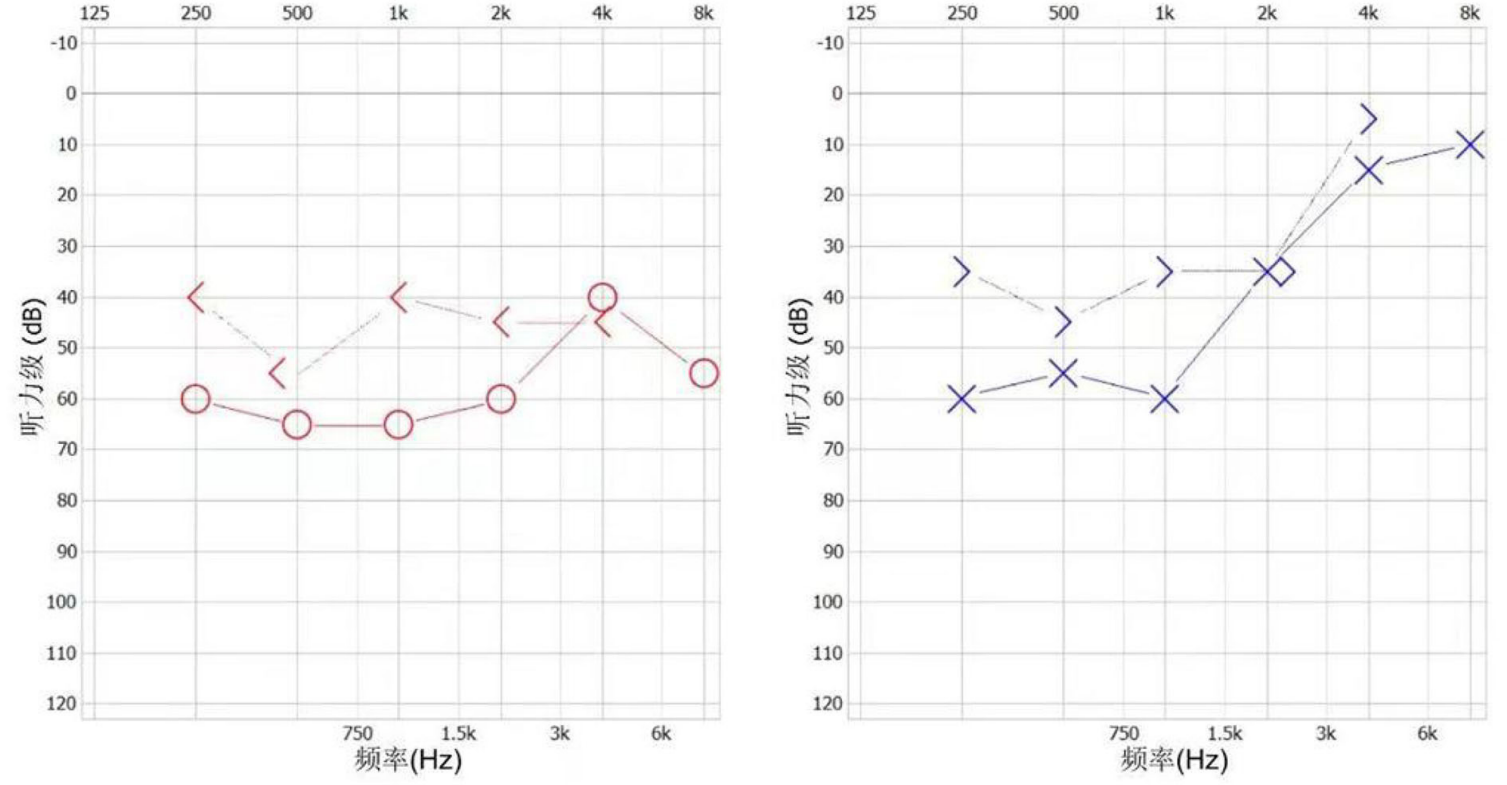
The patient's audiogram.

The patient presented with visual rotation, urinary dysfunction, nausea, and vomiting within a month post-discharge. Enlarged lesions involving the caudate nucleus and cortex were observed on brain MRI, particularly significant hyperintensity of foci on diffusion-weighted imaging (DWI). Therefore, PACNS was considered. The symptoms did not significantly improve upon treatment with immunoglobulin and rituximab (500 mg) to prevent relapse. Eventually, the patient was referred to the First Medical Center of Chinese PLA General Hospital. A review of his medical history revealed that the headache symptoms started 2 months pre-symptom-onset as intermittent pulsatile headaches in the right frontotemporal region. Physical examination showed a moderate cognitive decline (Mini-Mental State Examination score: 23/30, and Montreal Cognitive Assessment: 22/30). Other positive signs included pyramidal tract and meningeal irritation signs. Results of laboratory tests for routine blood parameters, biochemical factors, infection indicators, and tumor markers were normal. Furthermore, tests for demyelination-related antibodies, including those against AQP4, MOG, and GFAP, were negative. CSF findings showed 250 mm H_2_O initial pressure and 1,028.7 mg/L protein level. Brain MRI results indicated that the lesion had diminished, with multifocal enhancement, compared with the MRI finding 1 month before. Therefore, the possibility of PACNS disease was considered. However, the symptoms continued to progress, and. the patient complained of decreased vision. Ophthalmic examination showed poor vision (20/125 and 20/32 in the left and right eyes, respectively; Snellen scale) and optic-disc edema in both eyes and cotton-wool-like lesions in the retina of the right eye. The patient experienced a hearing decline in both ears 4 months after the onset. Subsequently, a hearing test showed binaural sensorineural deafness (low-medium -frequency) (Figure 2). A re-test of visual acuity (naked eye) revealed scores of 20/200 and 20/32 in the left and right eyes, respectively. Fundus fluorescein angiography displayed branch retinal artery occlusion and leakage of the micro-arterial wall (Figure 1). Ultimately, the diagnosis of SuS was confirmed based on the above findings (Figure 1), and subsequently, methylprednisolone and infliximab were administered. No recurrence of symptoms was observed, and cognition ability was normal (MMSE score: 30/30, and MoCA: 29/30) at the latest follow-up (01. Nov. 2022).

## Discussion

Susac syndrome is rare autoimmune microangiopathy that was first reported by Susac et al. in 1979 (3) and has an annual incidence of 0.024 per 100,000 (95% CI 0.010–0.047) (4). Only 13–30% of patients present with a full triad of vascular endothelium impairment in the brain, retina, and cochlea, and obstruction of small arteries during the initial stage (1). Thus, SuS may be easily misdiagnosed in the initial stage. In the present case, the patient showed rare clinical manifestations, including seizures and urinary dysfunction, as the initial symptoms, leading to misdiagnosis.

The diagnostic criteria established by the European Susac Association in 2016 include brain, retinal, and vestibulocochlear involvement (1). Although a central snowball-like lesion in the corpus callosum is a relatively characteristic image, it may not be present in the early stage, as was the case in our patient. He was initially misdiagnosed with ADEM because multiple lesions contributed to the white matter (particularly corpus-callosum and brainstem involvement), and he responded well to prednisone therapy. In the following stage, PACNS was diagnosed based on the following points: (1) The patient with the encephalopathy-like presentation; (2) Clinical manifestations are severe; (3) MRI show acute ischemia-like hyperintensity on DWI sequence (Figure 1). Distinguishing SuS from PACNS is challenging when the early manifestations are hemiparesis and epilepsy. Increased intracranial pressure and CSF protein level and lesions with restricted diffusion on DWI can be observed in both PACNS and SuS (5). Seizures seem more common in patients with PACNS than in patients with SuS (8/15 vs. 1/13, p = 0.035) according to a multicenter study (5). A total of 42% of patients with PACNS have multiple disseminated lesions including cortex, caudate, and deep white matter (6). Additionally, patients with SuS who manifested relapsing-remitting course with multiple periventricular T2 FLAIR hyperintensity may be easily misdiagnosed as multiple sclerosis (MS). A recent study found the diagnosis of MS was originally prioritized in 16% of patients with SuS (7). Nevertheless, for the present case, MS was not considered because encephalopathy-like episodes at the early stage rarely occur in patients with MS (8), especially with impairment in the caudate nucleus as well as significant hyperintensity of lesions on DWI. Unfortunately, the visual decline in the present case was considered to be caused by demyelination and intracranial hypertension. As the possible involvement of small retinal arteriopathy was ignored, fundus fluoroscopy was not performed in time, leading to an oversight in the differential diagnosis. Notably, SuS needs to be differentiated from lymphoproliferative disorders and specific infections. CSF examination, including evaluation of the inflammatory parameters and IL-10 levels and cytological assessment, can help with differential diagnosis.

Recently, the underlying pathogenesis of SuS is still far from clarification. An international multicenter study showed high-titer IgG1 and IgM anti-endothelial cell antibodies (AECA) in some patients with SuS, indicating that humoral autoimmunity may play a role in the pathogenesis of SuS (9). Patients with Sus were treated based on clinical experience. The latest treatment guidelines recommend stratified treatment according to the severity of the disease (1). For our patient, the symptoms improved after the administration of methylprednisolone combined with infliximab.

## Conclusion

Susac syndrome is often misdiagnosed owing to its rarity, especially in patients manifesting epilepsy, urinary dysfunction, and hemiparesis with or without cortical functional decline, as the initial manifestation. Therefore, brain MRI, binocular vision assessment, and hearing tests must be performed as early as possible when patients show these symptoms. Given the high sensitivity to suspicious clinical findings, regular follow-up is extremely crucial for a correct diagnosis.

## Data availability statement

The raw data supporting the conclusions of this article will be made available by the authors, without undue reservation.

## Ethics statement

The studies involving human participants were reviewed and approved by the First Medical Center of Chinese PLA General Hospital. The patients/participants provided their written informed consent to participate in this study. Written informed consent was obtained from the individual(s) for the publication of any potentially identifiable images or data included in this article.

## Author contributions

GW and WJ collected the data and prepared the manuscript. ZL analyzed the data and created the figures. LW and DH designed and supervised the work. All authors contributed to the article and approved the submitted version.

## References

[1] Marrodan M Fiol MP Correale J. Susac syndrome: challenges in the diagnosis and treatment. Brain. (2022) 145:858–71. 10.1093/brain/awab476 35136969

[2] Dörr J Krautwald S Wildemann B Jarius S Ringelstein M Duning T. Characteristics of Susac syndrome: a review of all reported cases. Nat Rev Neurol. (2013) 9:307–16. 10.1038/nrneurol.2013.82 23628737

[3] Susac JO Hardman JM Selhorst JB. Microangiopathy of the brain and retina. Neurology. (1979) 29:313–6. 10.1212/WNL.29.3.313 571975

[4] Seifert-Held T Langner-Wegscheider BJ Komposch M Simschitz P Franta C Teuchner B. Susac's syndrome: clinical course and epidemiology in a Central European population. Int J Neurosci. (2017) 127:776–80. 10.1080/00207454.2016.1254631 27788613

[5] Marrodan M Acosta JN Alessandro L Fernandez VC Contentti EC Arakaki N. Clinical and imaging features distinguishing Susac syndrome from primary angiitis of the central nervous system. J Neurol Sci. (2018) 395:29–34. 10.1016/j.jns.2018.09.029 30273791

[6] Boulouis G de Boysson H Zuber M Guillevin L Meary E Costalat V. Primary angiitis of the central nervous system: magnetic resonance imaging spectrum of parenchymal, meningeal, and vascular lesions at baseline. Stroke. (2017) 48:1248–55. 10.1161/STROKEAHA.116.016194 28330942

[7] Triplett JD Qiu J O'Brien B Gopinath S Trewin B Spring PJ. Diagnosis, differential diagnosis and misdiagnosis of Susac syndrome. Eur J Neurol. (2022) 29:1771–81. 10.1111/ene.15317 35262238 PMC9314104

[8] Buzzard KA Reddel SW Yiannikas C Riminton DS Barnett MH Hardy TA. Distinguishing Susac's syndrome from multiple sclerosis. J Neurol. (2015) 262:1613–21. 10.1007/s00415-014-7628-9 25547511

[9] Jarius S Kleffner I Dörr JM Sastre-Garriga J Illes Z Eggenberger E. Clinical, paraclinical and serological findings in Susac syndrome: an international multicenter study. J Neuroinflammat. (2014) 11:46. 10.1186/1742-2094-11-46 24606999 PMC3995917

